# How does news affect biopharma stock prices?: An event study

**DOI:** 10.1371/journal.pone.0296927

**Published:** 2024-01-26

**Authors:** Joonhyuk Cho, Manish Singh, Andrew W. Lo

**Affiliations:** 1 Laboratory for Financial Engineering, MIT, Cambridge, MA, United States of America; 2 Department of Electrical Engineering and Computer Science, MIT, Cambridge, MA, United States of America; 3 Computer Science and Artificial Intelligence Laboratory, MIT, Cambridge, MA, United States of America; 4 Operations Research Center, MIT, Cambridge, MA, United States of America; 5 Sloan School of Management, MIT, Cambridge, MA, United States of America; 6 Santa Fe Institute, Santa Fe, NM, United States of America; University of Durham: Durham University, UNITED KINGDOM

## Abstract

We investigate the impact of information on biopharmaceutical stock prices via an event study encompassing 503,107 news releases from 1,012 companies. We distinguish between pharmaceutical and biotechnology companies, and apply three asset pricing models to estimate their abnormal returns. Acquisition-related news yields the highest positive return, while drug-development setbacks trigger significant negative returns. We also find that biotechnology companies have larger means and standard deviations of abnormal returns, while the abnormal returns of pharmaceutical companies are influenced by more general financial news. To better understand the empirical properties of price movement dynamics, we regress abnormal returns on market capitalization and a sub-industry indicator variable to distinguish biotechnology and pharmaceutical companies, and find that biopharma companies with larger capitalization generally experience lower magnitude of abnormal returns in response to events. Using longer event windows, we show that news related to acquisitions and clinical trials are the sources of potential news leakage. We expect this study to provide valuable insights into how diverse news types affect market perceptions and stock valuations, particularly in the volatile and information-sensitive biopharmaceutical sector, thus aiding stakeholders in making informed investment and strategic decisions.

## Introduction

The biopharmaceutical industry is experiencing rapid growth due to the accelerated evolution of drug development, a result of such technological innovations as the CRISPR-Cas9 system [1], the mRNA vaccine toolkit [2], and the application of deep learning methods to drug development [3]. As the biopharmaceutical industry grows, investor interest in the biopharmaceutical sector is likewise increasing. Since the business model and the market reaction of the biopharmaceutical sector are distinct from other sectors, it is crucial to analyze its stocks separately from the rest of the market. For example, [4] found that pharmaceutical companies have outperformed the broader market in returns and Sharpe ratio, while biotechnology companies exhibit higher volatility and lower return compared to the market average. In light of these trends, [5] analyzed stock market reactions of pharmaceutical and biotechnology companies to the outcomes of clinical trials of candidate drugs.

The stock market is affected by multiple factors, including the fundamentals of the firm, such as its revenue, assets, and liabilities [6]; exchange rate fluctuations [7]; unexpected disasters such as terrorism [8] or pandemics [9]; announcements related to a company’s product [10]; mergers and acquisitions [11]; and other similar events or actions. One of the most credible, reliable, and immediate sources through which investors can find information about these factors is the business or financial news article. In an analysis of corporate press releases issued between April 2006 and August 2009, [12] found that announcements related to these market factors are accompanied by meaningful abnormal returns. A case study [13] even showed the significant impact of news articles on stock prices, exemplified by the case of EntreMed, where a New York Times article led to a dramatic and lasting increase in the company’s stock value, despite no new information being presented.

In this study, we clarify and analyze the historical impact of various news categories on biopharmaceutical companies, investigating the market’s response to each type of news. To do so, we conduct an event study on 1,012 biopharmaceutical companies using 503,107 news releases in 437 different news categories. To calculate abnormal returns with robustness, our study uses three different asset pricing models, the Fama–French five-factor model [14], the constant mean model [15], and the market model [16] as detailed in the Section: Abnormal Returns by News Categories. The event study shows that acquisition, product development, investment, regulation, earnings guidance, and analyst ratings significantly impact the stock prices of biopharmaceutical companies. The most positive stock returns are linked to acquisition-related news, especially for companies being acquired, while news about halted or failed product development results in major negative returns.

The rationale behind this comprehensive analysis is rooted in the need to understand and quantify the influence of diverse types of news on market perceptions and stock valuations in the biopharmaceutical sector. Characterized by high volatility [4] and information sensitivity [13], this sector provides an ideal context for examining the interplay between news and stock performance. By investigating the impact of news across a broad spectrum of categories, our study sheds light on the nuanced ways in which investors assimilate and react to information. Therefore, we expect this research to offer insights into the risk and return dynamics associated with different types of news, helping all stakeholders make more informed investment decisions and manage risk more effectively. In particular, biopharma companies can leverage these findings to better understand investor behavior, allowing them to engage in more effective communication and financial planning.

We begin by differentiating between pharmaceutical and biotechnology companies, and perform an event study to observe the functional differences between the two sectors. Even though these sectors both focus on drug development, they have different ways of developing treatments and therapeutics [17]. As a result, each sector also has distinct business models and financial characteristics [4, 18]. We therefore perform an event study for the two sectors, and compare their market reaction to news categories. Stock responses to news releases differ greatly between pharmaceutical and biotechnology companies, with biotech firms showing a larger magnitude of abnormal returns. While biotech stocks are particularly sensitive to acquisition news and negative product-related announcements, pharmaceutical stocks respond to a broader range of news, including equity actions, earnings, and dividends.

We also analyze the role of size by calculating the Pearson and Spearman correlation coefficients between the market capitalization and the abnormal return of the stocks in our study. In general, the market capitalization of a pharmaceutical company is higher than that of a biotechnology company. To clarify whether the abnormal return depends solely on market capitalization or its classification as a biotechnology or pharmaceutical company, we also run a two-factor ordinary least squares (OLS) regression with abnormal return as a dependent variable and market capitalization and company classification as independent variables. In examining the relationship between market capitalization and abnormal returns, especially concerning news categories like Acquisition, Clinical Trials, and Analyst Ratings, we find that companies with larger market capitalizations typically experience lower magnitudes of abnormal returns, irrespective of news sentiment. Regression analyses further confirm that market capitalization is the predominant factor affecting abnormal returns, with sub-industry distinctions playing a significant role in specific categories.

Finally, we perform our analysis not only for the date of a news release, but over an expanded window of time to detect provisional news leaks or surprise news about biopharmaceutical companies. Given that insider trading and news leaks are prevalent [19], our intent is to provide a foundation for further research into this phenomenon and to aid the further development of market abuse regulations for the biopharmaceutical industry. Our study finds that news related to acquisitions and product development intrigues abnormal returns even before the release of news, requiring further examination under market abuse regulations.

## Data preparation

We use two different datasets to investigate the impact of news releases on stock returns: the Dow Jones equities article dataset from RavenPack, and the daily U.S. stock returns dataset from the University of Chicago’s Center for Research in Security Prices (CRSP).

### Dow jones article data

We compile historical news articles data from RavenPack’s Dow Jones Edition dataset. This dataset includes news releases from multiple sources such as Dow Jones Financial Wires, the *Wall Street Journal*, *Barron’s*, and *MarketWatch*. Each news release uses RavenPack’s taxonomic system to assign the article to a group and category. The group is the higher-ranked taxonomic unit that classifies the theme of events (e.g., Acquisition-Mergers), while the category is a more detailed classification (e.g., Acquisition-Interest-Acquiree). In our dataset, there are 33 groups and 437 categories. To capture the earliest moment when the news is released to the public, we filtered the data to remove items with a novelty score of less than 100. The novelty score is provided by the Dow Jones Edition dataset. The earliest release of a news item receives a novelty score of 100, and lower novelty scores are assigned to later releases of duplicated news. Since a single event may have multiple news releases, this filtering criteria also eliminates duplicated articles. The categories in S1 Table are excluded from our analysis, since they are duplicated in other categories, or the news category itself is a review of the abnormal return event. Detailed descriptions of the categories included in this study can be found in Table S2 Table.

**Fig 1:**
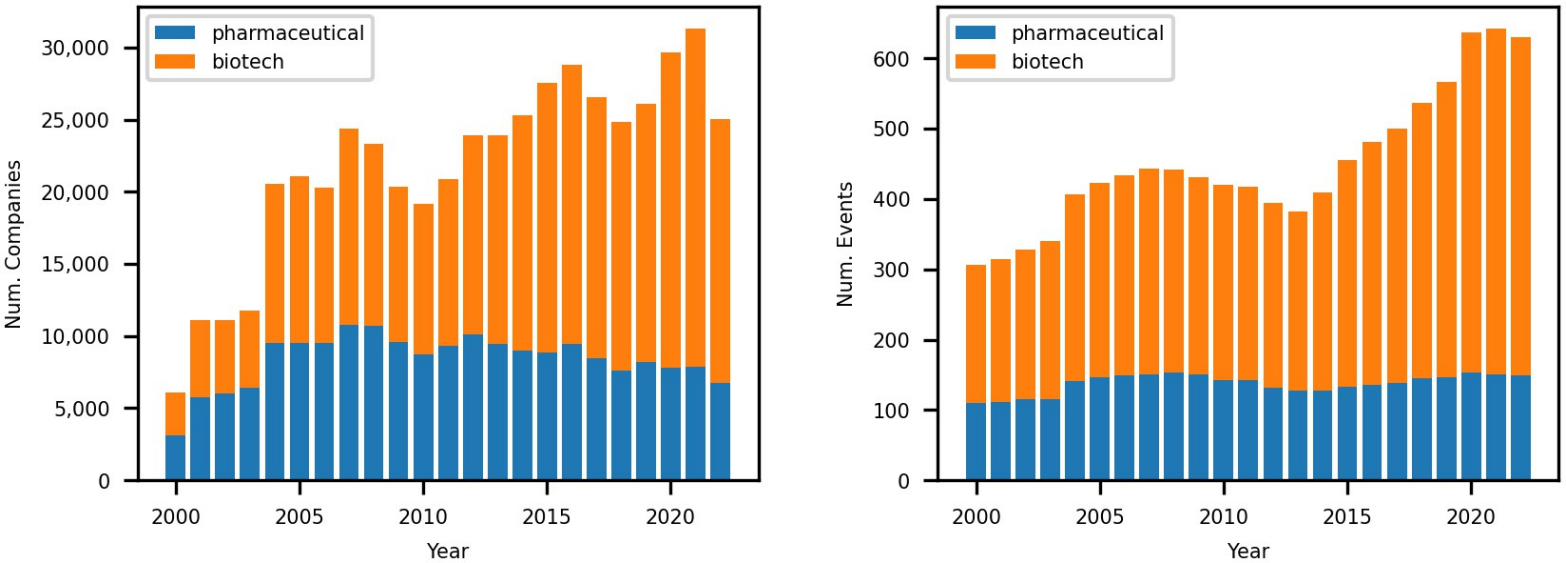
Time series number of the biopharma companies and their corresponding news articles between 2000 and 2022. Note that our news article data is from Jan 1st, 2000 to Oct 31st, 2022. This explains the temporal decrease in the number of news articles in 2022. Left: Time series number of the biopharma companies. Right: Time series number of the biopharma news articles.

To compare the characteristics of biotechnology versus pharmaceutical companies, we marked each news article with an additional identifier. A distinction is made between pharmaceutical and biotechnology companies using the GICS sub-industry code (35201010 for biotechnology companies and 35202010 for pharmaceutical companies). Our dataset comprises 310,540 articles from 731 biotechnology companies and 192,567 articles from 281 pharmaceutical companies between January 1, 2000, and October 31, 2022, for a total of 503,107 news releases from 1,012 different entities. The time series number of articles and companies is given in Fig 1. It is observed that the overall number of companies and articles has an increasing trend since 2000. Over the 21-year period covered by our dataset (2000 to 2021), the total number of biotechnology and pharmaceutical companies more than doubled (208%), and the annual number of articles increased over four-fold (485%). Separating the biotechnology and the pharmaceutical companies, we also observe that the biotechnology industry is the main contributor to the overall increase in the number of entities and events. While the number of pharmaceutical companies and their corresponding articles increased by 1.39 and 2.49 times, respectively, the number of biotechnology companies and their articles increased by 2.47 and 7.34 times, respectively. This steep increase is indicative of the rapid development of the biotechnology industry.

### Daily stock return data

To calculate the abnormal return caused by news releases, we use daily stock return data from the CRSP U.S. stock dataset. Like the news article data, we use only data from biotechnology and pharmaceutical companies by filtering by their GICS sub-industry code. Since the event study requires prior return data to calculate an abnormal return, additional return data is imported from 1999 to 2022 in order to correspond to the article data from 2000 to 2022. The article is linked to its corresponding return data through the matching table between the RavenPack entity ID and CUSIP number, and its abnormal return can then be calculated.

## Event study design

An event study is an empirical method for investigating the impact of an event on a security’s value, in this study, the stock price. By conducting an event study, we can quantify the market’s reaction to a specific type of event. In this paper, we perform an event study for each news article and news article category in our dataset on the daily stock return data of those companies classified as biopharmaceutical companies by GICS.

### Event date

In our study, the event date is defined as the date when the news article is first reflected in the market. If the news is released on a trading day before the market closes, the event date will be the same as the article’s release date. However, if the event happens after market close, we consider the event date to have taken place on the next trading day. Due to holidays and trading halts, there is a possibility that the immediate ‘next day’ of the news article is not a valid trading day. To resolve this problem, we search up to 5 calendar days after the event to find a valid ‘event date’, which must be a trading day. Once the event date is determined, the remaining event study uses ‘trading day’ rather than ‘calendar day’ as the unit of time.

### Abnormal return calculation

In our study, the Fama–French five-factor model [14] is used to estimate the expected and abnormal return in the event window of each news article release. The model estimates the expected return by incorporating size, value, profitability, and investment factors. The time series regression model is given as:

$$\begin{array}{c} R_{it}-R_{Ft}=\alpha_{i}+\beta_{i}\left(R_{Mt}-R_{Ft}\right)+s_{i}SMB_{t}+h_{i}HML_{t}+r_{i}RMW_{t}+c_{i}CMA_{t}+\epsilon_{it}\\ E\left[\epsilon_{it}\right]=0,Var\left[\epsilon_{it}\right]=\sigma_{\epsilon_{i}}^{2} \end{array}$$

where *R*_*it*_ is the expected return of stock *i* at date *t*, *R*_*Ft*_ is the risk-free return at date *t*, *SMB*_*t*_, *HML*_*t*_, *RMW*_*t*_ & *CMA*_*t*_ are factors based on size, value, operating profitability and investments, respectively, each at time *t*. The historical daily data are downloaded from the data library on Kenneth R. French’s website. (See https://mba.tuck.dartmouth.edu/pages/faculty/ken.french/data_library.html.) The abnormal return is determined by subtracting the expected return—as calculated by the regression model—from the actual return.

To verify the robustness of our analysis, we also use other models for our abnormal return calculation. First, the constant mean return model is applied to estimate the expected and abnormal returns [15]. The equation for the constant mean model is:

$$\begin{array}{c} R_{it}=\mu_{i}+\epsilon_{it}\\ E\left[\epsilon_{it}\right]=0,Var\left[\epsilon_{it}\right]=\sigma_{\epsilon_{i}}^{2} \end{array}$$

This model assumes that the stock return of an asset is independent and identically normally distributed over time, and therefore will have a constant mean and standard deviation. Hence, the expected return will be the average return of an asset during the estimation period.

Additionally, we apply the market model to the calculation of the abnormal return [16] as follows:

$$\begin{array}{c} R_{it}=\alpha_{i}+\beta_{i}R_{Mt}+\epsilon_{it}\\ E\left[\epsilon_{it}\right]=0,Var\left[\epsilon_{it}\right]=\sigma_{\epsilon_{i}}^{2} \end{array}$$

The market model is a simplified version of the factor model. It calculates the expected return by training a regression model to determine the intercept and slope coefficient, *α*_*i*_ and *β*_*i*_, respectively. We use the daily return of the value-weighted market portfolio of U.S. stocks from the CRSP database as the market return value, *R*_*Mt*_.

Since our study focuses on analyzing the impact of each news event *category* on the stock return, we aggregate the article data in each category. The abnormal return is then calculated as the difference between observed returns and returns estimated using the Fama–French five-factor model for each company on the specific event date. We use return data from 200 days before the event date to 30 days before the event date for the regression model, and capture the coefficients for each factor. To observe the abnormal return time series, we calculate the abnormal return from 5 days before the event date to 10 days after the event date. The aggregated abnormal return (AR) and its variance are then calculated by:

$$\begin{array}{c} AR_{category,t}=\frac{\sum_{i=1}^{n}AR_{event_{i},Company_{i},t}}{n}\\ Var\left(AR_{category,t}\right)=\frac{\sum_{i=1}^{n}Var\left(AR_{event_{i},Company_{i},t}\right)}{n^{2}} \end{array}$$

### Abnormal returns and market capitalization

To evaluate the relationship between the market capitalization of a company and its abnormal return, we first need to define the market capitalization on the event date. The market capitalization is calculated as follows:

$$\begin{array}{c} Market\;Cap_{Company,Date}=Price_{Company,Date-1,Closing}\times Shares_{Company} \end{array}$$

This calculation establishes a precise market capitalization for the company as of the day preceding the news announcement. This step is crucial for adjusting for any distortions in the event date’s abnormal returns, essentially debiasing their effect. We then calculate the relative market capitalization in order to take into account the effect of market growth by dividing the market capitalization by the S&P index.

$$\begin{array}{c} Relative\;Market\;Cap_{Company,Date}=\frac{Market\;Cap_{Company,Date}}{S\&P\;Index_{Date}} \end{array}$$

Since the difference in market capitalization between large pharmaceutical companies and small biotechnology companies is immense, (e.g., As of September 27th, 2023, the market capitalization of Pfizer Inc. (PFE) is over 180 billion USD, while the market capitalization of Aptose Biosciences Inc. (APTO) is around 180 million USD) we take the logarithm of each value to create a more linear fit to the abnormal return. This value, the adjusted market capitalization, is used for our analysis in the remainder of this study.

We next calculate the correlation coefficient between the abnormal return (computed by the Fama-French five-factor model) and the adjusted market capitalization. First, we choose five categories of news articles for biopharmaceutical companies based on their frequent appearance in the top positive and negative rankings for abnormal returns, highlighting their significance and influence on market behavior. We then use two different metrics to evaluate the correlation between market capitalization and abnormal return, the Pearson correlation coefficient [20] and Spearman’s rank correlation coefficient [21]. The Pearson correlation coefficient estimates the linear relationship between two variables, while Spearman’s rank correlation coefficient estimates the monotonic relationship between two variables [22].

However, because pharmaceutical companies tend to have a significantly higher market capitalization compared to biotechnology companies—which may result in spurious correlations—we run an additional OLS regression [23] with the abnormal return as a dependent variable and market capitalization and a pharmaceutical category indicator (0 if the firm is a biotechnology company, 1 if it is a pharmaceutical company) as independent variables. The main purpose of this OLS regression is to specify whether market capitalization or biopharmaceutical category is the dominant factor affecting the abnormal return.

### Information leaks and surprises

As an extension of our event study, we investigate possible candidates for news leaks or surprise news by comparing the cumulative abnormal return (CAR) of a stock before and after its associated news release. Past research has been used to detect leaks of judicial news by analyzing stock movement before and after judicial decisions [19]. Similarly, we compute the CAR for a two-day event window before and after a news release by summing the abnormal return each day as follows:

$$\begin{array}{c} CAR_{category,t_{1},t_{2}}=\sum_{t=t_{1}}^{t_{2}}AR_{category,t} \end{array}$$

where *AR*_*category*,0_ is the abnormal return of the category on the day of a news release.

In our study, we have chosen the

[-2,-1]

and

[0,+1]

event windows to effectively capture the immediate impact of news releases on stock returns. This selection acknowledges the potential asymmetry that can occur when news is released during trading hours. Despite this issue, we believe these windows are optimal for analyzing acute market responses, which are the primary focus of our study. We recognize the value in investigating alternate, more symmetric windows such as [+1, +2] in future research to gauge delays in market responses.

When there exists a statistically significant cumulative return (*p*-value under 0.05) before (*CAR*_*category*,−2,−1_) and after (*CAR*_*category*,0,1_) the release of a specific news category, we consider the category a candidate for leaked or surprise news. That is, if the sign of the abnormal return before the news is equal to the sign of the abnormal return after the news, the category is classified as a candidate for news leaks. If the signs are different from each other, however, the category is classified as a candidate for surprise news.

## Results and discussion

### Abnormal returns by news categories

The abnormal returns of different news categories are calculated using the Fama-French five-factor model, the constant mean model, and the market model. The most influential news categories are given in Table 1, with the most positive ones listed in Table 1a and the most negative ones listed in Table 1b. Categories that appeared fewer than 10 times or had lower than 95% statistical significance are filtered from Table 1.

**Table 1:** News categories and abnormal returns.

Group	Category	*AR*_*ff*_ (%)	*AR*_*cm*_ (%)	*AR*_*mm*_ (%)	Count
Acquisitions-Mergers	Acquisition-Scrutiny-Acquiree	28.81**	28.65**	28.73**	20
Acquisitions-Mergers	Acquisition-Acquiree	25.14**	25.24**	25.25**	564
Acquisitions-Mergers	Acquisition-Interest-Acquiree	10.74**	10.92**	10.87**	178
Acquisitions-Mergers	Acquisition-Rumor-Acquiree	9.35**	9.50**	9.45**	29
Acquisitions-Mergers	Merger	6.96**	7.01**	7.07**	342
Acquisitions-Mergers	Acquisition-Merger-Termination-Fee	6.98**	6.98**	6.99**	67
Product-Services	Fast-Track-Designation	5.79**	5.94**	5.92**	325
Earnings	EBITDA-Guidance-Up	5.62**	5.95**	5.97**	14
Acquisitions-Mergers	Acquisition-Bid-Rejected-Acquiree	4.69**	4.58**	4.50**	41
Product-Services	Clinical-Trials-Positive	4.49**	4.52**	4.49**	1,589
Dividends	Dividend-Guidance	4.47**	4.55**	4.41**	36
Analyst-Ratings	Analyst-Ratings-Positive	3.98**	4.01**	3.99**	2,602
Credit-Ratings	Credit-Rating-Watch-Positive	4.08**	3.76**	4.08**	25
Assets	Patent Filing	3.81**	3.94**	3.96**	110
Product-Services	Clinical-Trials-Complete	3.04*	3.34**	3.18**	262
(**a**) Categories with the 15 highest abnormal returns.					
Group	Category	*AR*_*ff*_ (%)	*AR*_*cm*_ (%)	*AR*_*mm*_ (%)	Count
Product-Services	Clinical-Trials-Negative	−13.61**	−13.54**	−13.67**	49
Product-Services	Product-Delayed	−11.73**	−12.81**	−12.54**	12
Product-Services	Product-Outage	−9.44**	−9.96**	−9.73**	28
Product-Services	Patient-Enrollment-Suspended	−9.62**	−9.49**	−9.69**	40
Earnings	Earnings-Guidance-Suspended	−9.33**	−9.46**	−9.69**	49
Product-Services	Clinical-Trials-Suspended	−9.33**	−9.63**	−9.23**	154
Product-Services	Product-Approval-Denied	−9.19**	−9.15**	−9.11**	99
Equity-Actions	Reverse-Stock-Splits	−8.81**	−8.72**	−8.75**	153
Equity-Actions	Bought-Deal	−8.09**	−7.90**	−8.61**	49
Equity-Actions	Reorganization	−6.67**	−6.84**	−6.70**	36
Analyst-Ratings	Analyst-Ratings-Negative	−6.08**	−6.15**	−6.11**	3,240
Product-Services	Product-Application-Withdrawn	−4.66**	−4.91**	−4.65**	71
Revenues	Revenue-Guidance-Down	−4.65**	−4.75**	−4.76**	284
Product-Services	Product-Discontinued	−4.42**	−4.33**	−4.38**	297
Earnings	Earnings-Guidance-Below-Expectations	−4.39**	−4.31**	−4.01**	14
(**b**) Categories with the 15 lowest abnormal returns.					

News categories associated with the (a) highest and (b) lowest abnormal returns after the release of news. Each AR is calculated using the Fama–French five-factor model (*AR*_*ff*_), the constant model (*AR*_*cm*_), and the market model (*AR*_*mm*_). The significance level is marked with asterisks (*: 95% confidence, **: 98% confidence). Each list is sorted by the average of abnormal returns calculated by the three different models.

It is notable that the top news categories with positive abnormal returns for a company are related to acquisitions and mergers, especially when the company is an acquiree. The finding that acquired entities benefit more than acquiring entities aligns with earlier studies investigating general acquisitions and mergers [24, 25]. Looking into the acquisitions and mergers sector, S1 Fig provides event study plots showing the success and failure of five different types of collaborations, including acquisitions, mergers, partnerships, and joint ventures. It shows that the success of collaboration brings a positive abnormal return on the release day in all five cases, in the order of acquisition (acquiree), merger, acquisition (acquirer), partnership, and joint venture. Negative news regarding collaboration also affects the return on the day of news release, especially for the acquiree, partnership, and joint venture cases. The significant difference in abnormal returns between acquiree and acquirer can be explained by the fact that an acquisition usually includes a premium that is paid by the acquiring company.

In contrast, the group with the most negative impact on a company’s stock is news related to product services, as shown in Table neg15. In other words, the halting of product development or product offerings is the most unfavorable factor for biotechnology and pharmaceutical companies. Event study plots of product-related categories are given in S2 Fig. News articles that contain information regarding negative clinical trial results, or the delay or discontinuation of a product create remarkable negative abnormal returns up to −13%. In comparison, positive news articles related to products, including information about positive clinical trial results or fast track designations create positive abnormal returns up to 6%, which are relatively small when compared to their negative counterparts.

In summary, the most optimistic news categories for stock returns of biopharmaceutical companies consist of articles related to acquisitions, especially for companies that are being acquired. Meanwhile, news stories about the failure or halt of product development carry with them significant negative abnormal returns. Other news categories such as changes in guidance, credit ratings, analyst ratings, and equity actions are also associated with statistically significant changes in the stock price of biopharmaceutical companies.

The observed disparities in stock responses to acquisition-related news—with a more pronounced effect for positive announcements (note: negative acquisition-related news, not detailed in Table 1, typically yield approximately −1% in abnormal returns), and to clinical trial-related news, more significantly impacted by negative outcomes—can be usefully interpreted within framework of the Efficient Market Hypothesis (EMH) [26]. The hypothesis suggests that stock prices incorporate all available information. Therefore, it is plausible that the market, adhering to the principles of EMH, preemptively adjusts for anticipated outcomes such as expected positive clinical trial results or potential drawbacks of negative acquisition news, thereby causing these events to have a less pronounced effect on stock prices once they actually occur.

### Biotech vs. pharma

As the stock market responses of biotechnology and pharmaceutical companies differ significantly from each other [4, 27], we separate our dataset into biotechnology and pharmaceutical companies, and perform event studies separately for each sector. The statistics of abnormal returns for pharmaceutical and biotechnology companies are shown in Table 2. In all cases—absolute, positive, and negative abnormal returns—the means and standard deviations for biotechnology firms are larger than those for pharmaceutical firms. This implies that biotechnology companies have greater sensitivity in their stock price compared to pharmaceutical companies, which aligns with other studies that have found that biotechnology companies inherently have greater volatility compared to pharmaceutical companies (e.g., [4]).

**Table 2:** Abnormal returns statistics: Pharmaceutical vs biotechnology.

	Total		Pharmaceutical		Biotechnology	
	Mean (%)	Std	Mean (%)	Std	Mean (%)	Std
Absolute AR	2.07	3.20	1.70	2.79	2.92	4.59
Positive AR	1.94	3.30	1.51	2.45	2.80	4.74
Negative AR	−2.24	3.08	−1.94	3.10	−3.05	4.40

Statistics of abnormal returns (AR) for pharmaceutical and biotechnology companies. Absolute AR statistics are calculated by obtaining the absolute value of the abnormal return.

The news categories with the most positive and negative abnormal returns by sub-industry are presented in Table 3. The comparative analyses of the top categories for positive and negative abnormal returns within pharmaceutical and biotechnology sectors—expanding on the data in Table 3—are detailed in S3 Table. In Table 3a and 3b, it is shown that news related to acquisitions and mergers has the highest positive abnormal return for both biotechnology and pharmaceutical companies. However, the amount of abnormal return is up to 4 times larger for biotechnology companies compared to pharmaceutical companies, even within the same category of news. The extremely high abnormal return for biotechnology companies being acquired can be explained by the fact that acquisition is one of the main exit strategies for small biotech companies [28].

**Table 3:** News categories and abnormal returns for pharmaceutical and biotechnology companies.

Group	Category	AR (%)
Acquisition-Mergers	Acquisition-Acquiree	10.09**
Acquisition-Mergers	Acquisition-Interest-Acquiree	8.21**
Credit-Ratings	Credit-Rating-Watch-Positive	7.18**
Dividends	Dividend-Guidance	4.57**
Acquisition-Mergers	Acquisition-Rumor-Acquiree	4.41**
Acquisition-Mergers	Acquisition-Bid-Rejected-Acquiree	3.74**
Product-Services	Clinical-Trials-Complete	3.72**
Acquisition-Mergers	Merger	3.39**
Product-Services	Clinical-Trials-Positive	3.30**
Regulatory	Regulatory-Investigation-Completed	3.28*
(**a**) Categories with the 10 highest abnormal returns (Pharmaceutical Companies)		
Group	Category	AR (%)
Acquisition-Mergers	Acquisition-Acquiree	39.26**
Acquisition-Mergers	Acquisition-Scrutiny-Acquiree	34.72**
Acquisition-Mergers	Acquisition-Rumor-Acquiree	15.44**
Acquisition-Mergers	Acquisition-Merger-Termination-Fee	13.80**
Acquisition-Mergers	Acquisition-Interest-Acquiree	13.26**
Acquisition-Mergers	Acquisition-Completed-Acquiree	11.55**
Acquisition-Mergers	Merger	10.29**
Product-Services	Fast-Track-Designation	6.65**
Assets	Facility-Relocation	6.12*
Assets	Patent-Filing	5.85**
(**b**) Categories with the 10 highest abnormal returns (Biotechnology Companies)		
Group	Category	AR (%)
Equity-Actions	Reverse-Stock-Splits	−10.63**
Product-Services	Patient-Enrollment-Suspended	−8.81**
Earnings	Earnings-Guidance-Suspended	−6.57**
Product-Services	Product-Approval-Denied	−5.81**
Analyst-Ratings	Analyst-Ratings-Negative	−4.66**
Product-Services	Clinical-Trials-Negative	−4.29**
Revenues	Revenue-Guidance-Down	−3.83**
Product-Services	Product-Outage	−3.33**
Equity-Actions	Expenses-Guidance-Down	−3.25**
Earnings	Operating-Earnings-Negative	−2.73**
(**c**) Categories with the 10 lowest abnormal returns (Pharmaceutical Companies)		
Group	Category	AR (%)
Product-Services	Clinical-Trials-Negative	−26.03**
Product-Services	Product-Outage	−18.88**
Product-Services	Product-Approval-Denied	−16.26**
Product-Services	Clinical-Trials-Suspended	−14.03**
Earnings	Earnings-Guidance-Suspended	−13.68**
Product-Services	Product-Application-Withdrawn	−10.42**
Product-Services	Patient-Enrollment-Suspended	−10.00**
Equity-Actions	Bought-Deal	−9.54*
Product-Services	Product-Discontinued	−9.03**
Equity-Actions	Reorganization	−8.26**
(**d**) Categories with the 10 lowest abnormal returns (Biotechnology Companies)		

News categories associated with the highest and lowest abnormal returns after the release of the news related to pharmaceutical (panels a and c, respectively) and biotechnology (panels b and d, respectively) companies. Abnormal return is calculated by the Fama-French five-factor model. The significance level is marked with asterisks (*: 95% confidence, **: 98% confidence).

The differences in negative news categories between the biotechnology and pharmaceutical sectors are more obvious. Comparing Table 3c and 3d, the clear difference between the sectors is that biotechnology companies are more sensitive to negative news about their products than pharmaceutical companies. Since the primary objective of biotechnology companies is successful drug development and exit or acquisition (see [28, 29]), news about failure or the suspension of further development is very detrimental for companies in that sector. However, the business model of a pharmaceutical company does not generally depend on a single product’s performance. As a result, we observe that news about equity actions and earnings are as important as product-related news for pharmaceutical companies in Table 3c.

In summary, we observe a significant difference in stock responses to news releases between pharmaceutical and biotechnology companies, with biotechnology companies showing a larger magnitude of abnormal returns than pharmaceutical companies. The most influential news categories were also different between the two sectors. Stocks of biotechnology companies were more sensitive to positive news about acquisitions and mergers and negative product-related announcements. However, pharmaceutical stocks are affected by a wider variety of categories, including news about equity actions, earnings, and dividends.

### Abnormal returns and market capitalization

Given the prevalence and magnitude of their abnormal returns, we study the relationship between market capitalization and abnormal returns for three groups of news categories: Acquisition, Clinical Trials, and Analyst Ratings.

**Fig 2:**
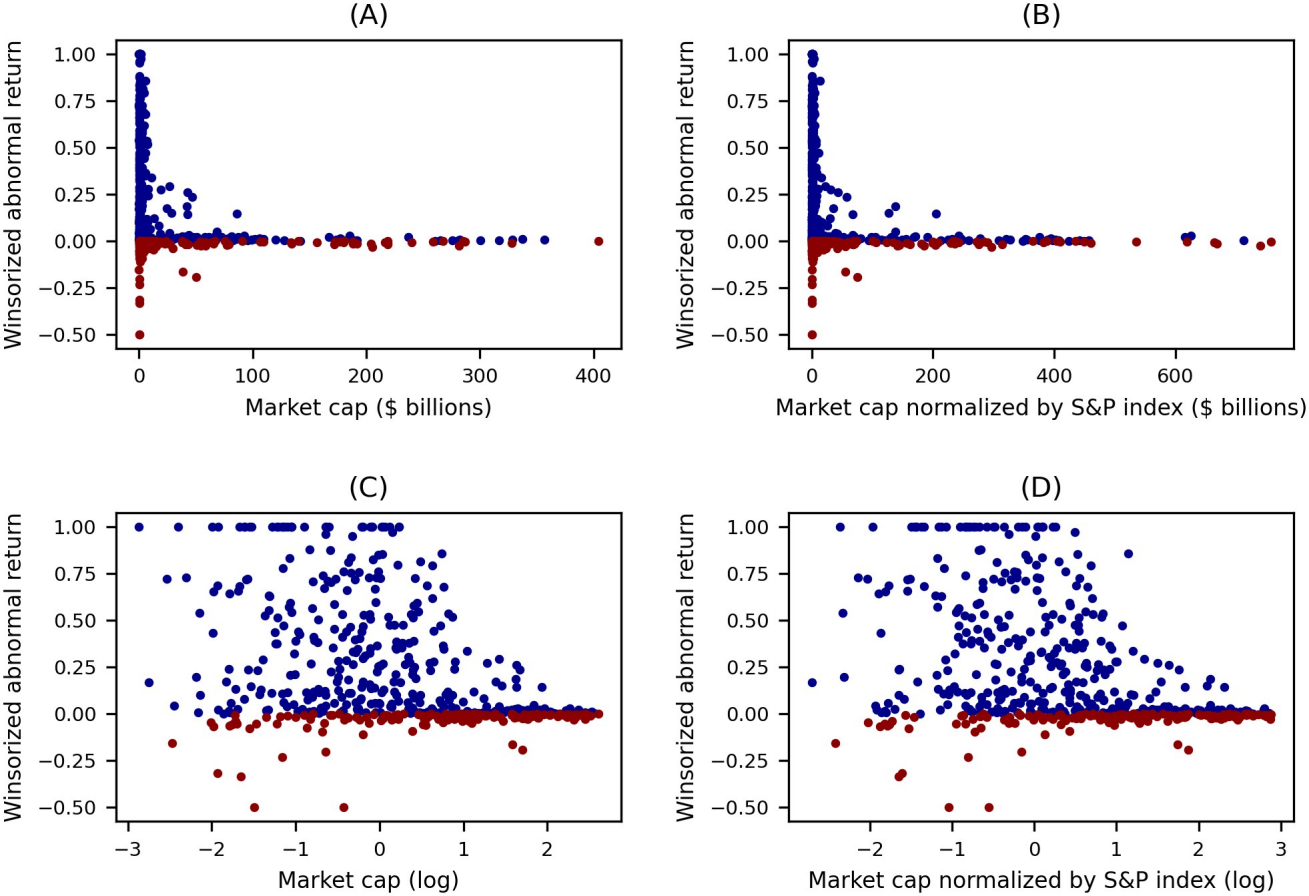
Scatter plots of market capitalization and abnormal returns with Acquisition-Acquiree news. Blue dots are positive abnormal returns and red dots are negative abnormal returns. Each abnormal return is winsorized to the range of [−0.5, 1] for better visualization. Different market capitalizations are used in different graphs, (A): Original market cap (Pearson Coefficient: −0.27, *p* = 0.00, Spearman’s Coefficient: −0.43, *p* = 0.00), (B): Market cap normalized by S&P index (Pearson Coefficient: −0.27, *p* = 0.00, Spearman’s Coefficient: −0.44, *p* = 0.00), (C): Logarithm of market cap (Pearson Coefficient: −0.42, *p* = 0.00, Spearman’s Coefficient: −0.43, *p* = 0.00), (D): Logarithm of market cap normalized by S&P index. (Adjusted market cap) (Pearson Coefficient: −0.43, *p* = 0.00, Spearman’s Coefficient: −0.44, *p* = 0.00).

For the news category of Acquisition, Fig 2 shows scatter plots of market capitalization versus abnormal returns at the time of acquisition news of the acquired companies. We observe negative correlation coefficients, implying there is a negative relationship between market capitalization and abnormal returns. Since the market capitalization ranges from hundreds of millions to hundreds of billions of U.S. dollars, we take the logarithm of the market capitalization to provide a more linear relationship between the market capitalization and the abnormal return. (This can be seen comparing the Pearson correlation coefficient in Fig 2(a), −0.27, to that in Fig 2(c), −0.43.) The Spearman’s rank correlation coefficient is not affected by this transformation since the rank of the market capitalization will not change after taking its logarithm. (This can be seen comparing the Spearman’s correlation coefficient in Fig 2(a), −0.43, to that in Fig 2(c), −0.43.) To investigate this linear correlation while compensating for the effect of market growth over time, we use the logarithm of market capitalization divided by the S&P Index as the independent variable, which we term the adjusted market capitalization.

Expanding this approach, Table 4 shows the Pearson and Spearman’s correlation coefficients between adjusted market capitalization and abnormal returns to different categories of news. It is shown that positive news categories (Acquisition-Acquiree, Clinical-Trials-Positive, and Analyst-Ratings-Positive) have negative correlations, while negative news (Clinical-Trials-Negative and Analyst-Ratings-Negative) has a positive correlation. This implies that the companies with a larger market capitalization tend to experience a lower magnitude of abnormal returns, regardless of the nuance of the news. As seen in Table 4, news regarding negative clinical trial results has the highest magnitude of correlation, over 0.5, followed by news about acquired companies, with a correlation magnitude of over 0.4.

**Table 4:** Pearson and spearman’s correlation coefficients.

Group	Category	Pearson (*p*-value)	Spearman (*p*-value)	Count
Acquisitions-Mergers	Acquisition-Acquiree	−0.428 (0.00)	−0.440 (0.00)	564
Clinical-Trials	Clinical-Trials-Positive	−0.148 (0.00)	−0.112 (0.00)	1,589
Clinical-Trials	Clinical-Trials-Negative	0.575 (0.00)	0.519 (0.00)	49
Analyst-Ratings	Analyst-Ratings-Positive	−0.214 (0.00)	−0.255 (0.00)	2,602
Analyst-Ratings	Analyst-Ratings-Negative	0.210 (0.00)	0.271 (0.00)	3,240

Pearson and Spearman’s correlation coefficients between adjusted market capitalization and abnormal returns for the selected categories and the news count within the category.

The observed correlation between market capitalization and the magnitude of abnormal returns following clinical trial news may be attributed to the breadth of drug development portfolios in larger biopharmaceutical companies. Such entities typically manage drug candidates across a range of development stages, and also have several commercialized drugs [30], which greatly reduces risk. In contrast, smaller companies often have a much smaller number of developmental drugs and may lack any commercialized products [31], making their valuation more susceptible to the outcomes of individual clinical trials. Therefore, a single clinical trial’s results tend to have a disproportionally larger impact on the stock price of smaller firms than on their larger counterparts.

Finally, the results of our OLS regression between abnormal returns as a dependent variable, and the market capitalization and sub-industry indicator (0 if a biotechnology company, 1 if a pharmaceutical company) as independent variables are stated in Table 5. To compare their impact on the abnormal return, the market capitalization and the sub-industry indicator variables are both standardized to a mean of 0 and a standard deviation of 1. These results show that market capitalization has a statistically significant (*p*-value < 0.05) greater correlation with abnormal returns in all categories of interest. The sub-industry indicator affects abnormal returns significantly in certain categories, but, notably, not in Clinical-Trials-Positive or Analyst-Ratings-Negative. By analyzing the OLS results, it is clear that market capitalization is the main factor, with a statistically significant negative correlation with the magnitude of abnormal returns, and that the sub-industry indicator only has a significant negative correlation with the magnitude of abnormal returns for certain categories. This result aligns with the conclusion from Pearson and Spearman’s correlation analysis.

**Table 5:** Summary of OLS regression.

Group	Category	Adjusted Market Cap (*p*-value)	Bio/Pharma Indicator (*p*-value)
Acquisitions-Mergers	Acquisition-Acquiree	−0.168 (0.00)	−0.078 (0.00)
Clinical-Trials	Clinical-Trials-Positive	−0.030 (0.00)	−0.001 (0.89)
Clinical-Trials	Clinical-Trials-Negative	0.126 (0.01)	0.069 (0.04)
Analyst-Ratings	Analyst-Ratings-Positive	−0.022 (0.00)	−0.005 (0.03)
Analyst-Ratings	Analyst-Ratings-Negative	0.032 (0.00)	0.005 (0.07)

Regression slope and *p*-value of OLS with X of adjusted market capitalization, pharmaceutical indicator (0 if biotechnology company, 1 if pharmaceutical company) and Y of abnormal returns.

### Information leaks and surprises

We also examine our dataset for possible candidates of stocks in which abnormal returns are associated with information leaks or surprise news. These candidates are detected by performing an event study using windows at varying time lengths. Table 6 shows the CAR of our candidate categories and the corresponding *p*-values for leaks and surprise candidates.

**Table 6:** News categories for potential leakage and surprise news.

Category	*CAR*_−2,−1_	*CAR*_0,1_	*p*_−2,−1_	*p*_0,1_	Count
Acquisition-Scrutiny-Acquiree*	29.5	27.1	0.00	0.00	20
Acquisition-Acquiree	1.5	25.3	0.01	0.00	564
Acquisition-Interest-Acquiree**	2.7	10.9	0.00	0.00	178
Acquisition-Rumor-Acquiree	3.6	10.3	0.00	0.00	29
Acquisition-Merger-Termination-Fee*	7.5	6.5	0.00	0.00	67
Analyst-Ratings-Positive**	1.1	4.2	0.00	0.00	2,602
Private-Placement	1.4	3.4	0.00	0.00	411
Credit-Rating-Watch-Positive	2.5	3.1	0.00	0.00	25
Price-Target-Upgrade*	3.8	2.7	0.00	0.00	3,289
Acquisition-Completed-Acquiree**	2.0	2.3	0.00	0.00	63
(**a**) Candidates for positive news leakage					
Category	*CAR*_−2,−1_	*CAR*_0,1_	*p*_−2,−1_	*p*_0,1_	Count
Clinical-Trials-Negative	−1.2	−13.0	0.00	0.00	49
Product-Approval-Denied	−1.9	−10.4	0.01	0.00	99
Earnings-Guidance-Suspended	−2.0	−9.7	0.00	0.00	49
Debt-Increase	−2.1	−3.2	0.00	0.00	28
Executive-Firing*	−2.3	−3.2	0.00	0.00	38
Price-Target-Downgrade*	−2.8	−2.8	0.04	0.01	3,200
Fraud-Defendant**	−1.7	−2.8	0.00	0.00	118
Credit-Rating-Watch-Negative	−1.2	−2.6	0.00	0.00	74
(**b**) Candidates for negative news leakage					
Category	*CAR*_−2,−1_	*CAR*_0,1_	*p*_−2,−1_	*p*_0,1_	Count
EBITDA-Negative	6.8	−7.4	0.00	0.02	12
Partnership-Terminated	7.1	−3.0	0.00	0.00	123
Company-For-Sale	6.5	−1.7	0.00	0.02	14
(**c**) Candidates for negative surprise news					

Cumulative Abnormal Return (CAR, %), *p*-value, and number of news items in the candidate categories of (a) positive news leakage, (b) negative news leakage, and (c) positive surprise news. If more than 10% of news items are followed by the same group (e.g. Acquisitions-Mergers) news within four trading days, the category name is marked with *. If more than 10% of news items are followed by the same group news within 14 trading days, the category name is marked with **.

Table 6a and 6b give our candidate categories for positive and negative news leaks, respectively. Some candidates—e.g., price targets or analyst rating news items—can be rejected by interpreting them as the consequence of causality. In other words, the trend of the stock price may affect analyst opinion and thus induce a news release. However, insider information related to acquisitions or products has a greater possibility of being an actual news leaks, since any abnormal return before the news release would not cause the release of that news. This is especially the case for acquisitions, since the news categories related to acquisitions have the largest impact on the abnormal return of biopharmaceutical companies, as shown in Section: Abnormal Returns by News Categories. Further study is required for market abuse regulation to verify whether these news categories might provide additional evidence of malicious information leaks.

Not all of these events are necessarily malign. Our reasoning is that if the same type (especially, same news group) of event happened frequently in the near past, it may affect the CAR in the earlier window (*CAR*_−2,−1_). Therefore, we also examined the categories in which more than 10% of the news was released in four trading days and marked them with *, and similarly those categories in which more than 10% of the news was released in 14 trading days, and marked them with **. These categories are less likely to be instances of information leaks, since the identical category of news recurred in a number of cases.

The news categories in Table 6c are candidates for negative surprise, in which a stock experienced positive abnormal returns before the news release and negative abnormal returns after the release. The categories in Table 6c are likely more secure in their identification than the categories in Table 6a or 6b.

## Conclusion

In this paper, we analyze the impact of various categories of news on U.S. biotechnology and pharmaceutical companies by performing event studies using RavenPack news article data and CRSP daily stock return data. We observe that acquisition-related news—especially news about a company being acquired—brings the most positive abnormal return to the company. On the negative side, a setback in the drug development process results in significant negative abnormal returns. The evidence suggests that acquisition events and drug development outcomes are pivotal determinants of stock valuation in the biopharmaceutical sector.

By comparing the abnormal return statistics between biotechnology and pharmaceutical companies, we determined that the mean and standard deviation of abnormal returns are larger in biotechnology companies than in pharmaceutical companies. Biotechnology stocks are strongly affected by positive acquisition news and negative product-related news. However, pharmaceutical stocks are also affected by general financial news in addition to these other news categories. We also perform a regression analysis to determine whether any correlation exists between market capitalization and abnormal return. Companies with larger market capitalization tend to experience a smaller degree of abnormal returns compared to smaller companies. By running a regression analysis using market capitalization and a sub-industry indicator as the independent variables, we show that both market capitalization and the type of sub-industry of a firm (i.e., biotechnology or pharmaceutical) are statistically significant factors, although market capitalization is more highly correlated. In addition, we use our dataset to uncover candidates for news leaks and surprise news by examining CARs within news categories using broader observation windows.

## Supporting information

S1 Table Delisted categories in our analysis and the reason for delisting. (PDF)

S2 Table News categories included in the analysis and their descriptions. (PDF)

S3 Table Comparative analysis of Average Abnormal Return (AAR) for top positive/negative Abnormal Return (AR) Categories in pharmaceutical and biotechnology sectors based on Table 3 data. Side-by-side comparison of average abnormal returns (AAR) for the pharmaceutical and biotechnology sectors across the top categories from Table 3, that elicited (a) positive, (b) negative market reactions. Differences in AR are calculated by subtracting pharmaceutical values from biotechnology counterparts. The significance of these differences is determined using a Welch’s T-test, with p-values (one-tailed) indicating the likelihood of the results occurring by chance. Categories where the p-value exceeds 5% are marked in red to denote lower statistical significance.
(PDF)

S4 Table (CSV)

S5 Table (CSV)

S6 Table (CSV)

S7 Table (CSV)

S8 Table (CSV)

S1 Fig Event study plots for the success and failure of collaboration-related news. By broad category, they are (A),(B): acquisition-acquiree, (C),(D): acquisition-acquirer, (E),(F): merger, (G),(H): partnership, and (I),(J): joint-venture.
(PDF)

S2 Fig Event study plots for Product-services related news. By broad category, they are (A),(B): clinical-trials, (C): fast-track-designation, and (D),(E),(F): product.
(PDF)
