# Supplementary material for: How does news affect biopharma stock prices?: An event study
Source: PLoS One. 2024 Jan 26;19(1):e0296927. doi: 10.1371/journal.pone.0296927 (PMC10817120; doi:10.1371/journal.pone.0296927)
Supplement: S1 Table — (PDF) [file pone.0296927.s001.pdf]

Supporting Information

Table S1. Delisted Categories in Our Analysis and the Reason for Delisting

Group	Category	Reason for Delisting
Stock-Prices	Stock-Gain	Directly related to return
Stock-Prices	Stock-Loss	Directly related to return
Order-Imbalances	Sell-Imbalance	Directly related to return
Order-Imbalances	Buy-Imbalance	Directly related to return
Order-Imbalances	Delay-Imbalance	Directly related to return
Order-Imbalances	Mkt-Open-Sell-Imbalance	Directly related to return
Order-Imbalances	Mkt-Open-Buy-Imbalance	Directly related to return
Order-Imbalances	Mkt-Close-Sell-Imbalance	Directly related to return
Order-Imbalances	Mkt-Close-Buy-Imbalance	Directly related to return
Analyst-Ratings	Analyst-Ratings-History-Positive	Duplicated with Analyst-Ratings-Negative
Analyst-Ratings	Analyst-Ratings-History-Neutral	Duplicated with Analyst-Ratings-Neutral
Analyst-Ratings	Analyst-Ratings-History-Negative	Duplicated with Analyst-Ratings-Positive
Equity-Actions	Trading-Resumed	Problem with calculating abnormal return
Equity-Actions	Trading-Halt	Problem with calculating abnormal return
Earnings	Earnings-Per-Share-Above-Expectations	Duplicated with Earnings-Above-Expectations
Earnings	Earnings-Per-Share-Below-Expectations	Duplicated with Earnings-Below-Expectations
Earnings	Earnings-Per-Share-Meet-Expectations	Duplicated with Earnings-Meet-Expectations
Earnings	Earnings-Per-Share-Positive	Duplicated with Earnings-Positive
Earnings	Earnings-Per-Share-Negative	Duplicated with Earnings-Negative
Earnings	Earnings-Per-Share-Up	Duplicated with Earnings-Up
Earnings	Earnings-Per-Share-Down	Duplicated with Earnings-Down
Earnings	Earnings-Per-Share	Duplicated with Earnings
Earnings	Earnings-Per-Share-Guidance	Duplicated with Earnings-Guidance
