# Supplementary material for: How does news affect biopharma stock prices?: An event study
Source: PLoS One. 2024 Jan 26;19(1):e0296927. doi: 10.1371/journal.pone.0296927 (PMC10817120; doi:10.1371/journal.pone.0296927)
Supplement: S2 Table — (PDF) [file pone.0296927.s002.pdf]

Supporting Information

Table S2. News Categories included in the Analysis and Their Descriptions

Category	Description
Acquisition-Acquiree	Acquisition news with the acquired company.
Acquisition-Acquirer	Acquisition news with the acquiring company.
Acquisition-Bid-Rejected-Acquiree	News related to the rejection of acquisition bid from the acquired company's side.
Acquisition-Completed-Acquiree	Acquisition news (completed acquisition) with the acquired company.
Acquisition-Failed-Acquiree	Failed acquisition news with the acquired company.
Acquisition-Failed-Acquirer	Failed acquisition news with the acquiring company.
Acquisition-Interest-Acquiree	Acquisition interest news with the acquired company.
Acquisition-Merger-Termination-Fee	Termination fee for merger or acquisition. This also implies the compensation regarding breakup of merger or acquisition.
Acquisition-Rumor-Acquirer	Acquisition rumor news with the acquiring company.
Acquisition-Rumor-Acquiree	Acquisition rumor news with the acquired company.
Acquisition-Scrutiny-Acquiree	Acquisition scrutiny news with the acquired company.
Analyst-Ratings-Negative	Negative change in the analyst's ratings of the company's stock.
Analyst-Ratings-Positive	Positive change in the analyst's ratings of the company's stock.
Bought-Deal	An announcement that a financial institution has already purchased a specified number of securities from a company.
Clinical-Trials-Complete	Completion of the company's clinical trial.
Clinical-Trials-Negative	Negative results of the company's clinical trial.
Clinical-Trials-Positive	Positive results of the company's clinical trial.
Clinical-Trials-Suspended	Suspension of the company's clinical trial.
Company-For-Sale	Announcement that a business is available for purchase, either in part or as a whole, by potential buyers or investors.
Credit-Rating-Watch-Positive	Credit rating agency considering an upgrade in a company's credit rating due to improved financial prospects
Debt-Increase	Increase in the company's debt.
Dividend-Guidance	Guidance news about the company's dividend.
Earnings-Guidance-Below-Expectations	The company's earning guidance below expectations.
Earnings-Guidance-Suspended	Temporal discontinuation of providing earnings forecasts or estimates to the public.
EBITDA-Guidance-Up	Increase in the company's EBITDA guidance.
EBITDA-Negative	Negative news with the company's EBITDA.

Executive-Firing	Termination or dismissal of a high-level corporate executive from their position within a company.
Expenses-Guidance-Down	Decrease in the company's expenses guidance.
Facility-Relocation	Moving a business or organization's physical operations, such as offices or production facilities.
Fast-Track-Designation	The company's decision to pursue fast track designation for the drug.
Fraud-Defendant	An individual or entity accused of participating in fraudulent activities and facing legal action or charges.
Merger	Merger-related news.
Merger-Failed	Merger failure related news.
Joint-Venture	Business arrangement where two or more companies collaborate to create a new entity or project.
Joint-Venture-Terminated	Conclusion or dissolution of a collaborative business arrangement.
Operating-Earnings-Negative	Decrease in the company's operation earnings.
Partnership	News regarding business relationship in which two or more entities collaborate.
Partnership-Terminated	Conclusion or dissolution of a business arrangement between two or more parties who were previously in a partnership.
Patent-Filing	Submission of an application to a government authority seeking the protection of a new invention or innovation.
Patient-Enrollment-Suspended	Temporary halt in the process of enrolling individuals in a clinical trial or medical study.
Price-Target-Downgrade	Decrease in price target for the company's stock.
Price-Target-Upgrade	Increase in price target for the company's stock .
Private-Placement	Sale of securities to a select group of investors.
Product-Application-Withdrawn	Voluntary removal of an application of regulatory approval or licensing of a product (drug).
Product-Approval-Denied	Rejection of an application for the approval of a product (drug) by regulatory authorities
Product-Delayed	Delay of the company's product (drug) release or development
Product-Discontinued	Discontinuation of the company's product (drug)
Product-Outage	The company's product (drug) suffering from a product outage.
Product-Recall	Process of removing or withdrawing a product from the market due to safety concerns, defects, or regulatory non-compliance.
Product-Release	Launch or introduction of a new product into the market.
Regulatory-Investigation-Completed	Completion of the regulatory investigation.
Reorganization	The process of restructuring a company's operations, management, or ownership to improve efficiency or adapt to new circumstances.

Revenue-Guidance-Down	Decrease in the company's revenue guidance.
Reverse-Stock-Splits	Corporate actions that reduce the number of outstanding shares per stakeholder, often to increase the stock's price.
Savings-Guidance	Guidance news about the company's savings.
