# Supplementary material for: How does news affect biopharma stock prices?: An event study
Source: PLoS One. 2024 Jan 26;19(1):e0296927. doi: 10.1371/journal.pone.0296927 (PMC10817120; doi:10.1371/journal.pone.0296927)
Supplement: S3 Table — Side-by-side comparison of average abnormal returns (AAR) for the pharmaceutical and biotechnology sectors across the top categories from Table 3, that elicited (a) positive, (b) negative market reactions. Differences in AR are calculated by subtracting pharmaceutical values from biotechnology counterparts. The significance of these differences is determined using a Welch’s T-test, with p-values (one-tailed) indicating the likelihood of the results occurring by chance. Categories where the p-value exceeds 5% are marked in red to denote lower statistical significance. (PDF) [file pone.0296927.s003.pdf]

Supporting Information

Table S3 (a). Comparative Analysis of Average Abnormal Return (AAR) for Top Negative Abnormal Return (AR) Categories in Pharmaceutical and Biotechnology Sectors Based on Table 3 Data

	Pharmaceutical		Biotechnology		Difference	
Category	AAR (%)	Var	AAR (%)	Var	AR (%)	P-Value
Acquisition-Acquiree	10.09	1.98e-3	39.26	3.08e-3	29.17	0.00
Acquisition-Bid-Rejected-Acquiree	3.74	4.47e-3	5.51	7.76e-3	1.77	0.23
Acquisition-Completed-Acquiree	-0.66	3.06e-3	11.55	9.42e-3	12.21	0.00
Acquisition-Interest-Acquiree	8.21	3.55e-3	13.26	5.52e-3	5.05	0.00
Acquisition-Merger-Termination-Fee	2.08	5.26e-3	13.80	7.66e-3	11.72	0.00
Acquisition-Scrutiny-Acquiree	-4.73	2.35e-2	34.72	1.65e-2	39.45	0.03
Acquisition-Rumor-Acquiree	4.41	4.50e-3	15.44	1.94e-2	11.03	0.01
Clinical-Trials-Complete	3.72	5.12e-3	2.53	4.23e-3	-1.19	0.08
Clinical-Trials-Positive	3.30	1.72e-3	5.05	1.47e-3	1.75	0.00
Credit-Rating-Watch-Positive	7.18	5.70e-3	0.14	8.94e-3	-7.04	0.03
Dividend-Guidance	4.57	3.44e-3	3.97	1.31e-2	-0.60	0.45
Facility-Relocation	-1.24	7.43e-3	6.12	1.57e-2	7.36	0.06
Fast-Track-Designation	2.50	4.55e-3	6.65	3.22e-3	4.15	0.00
Merger	3.39	4.06e-3	10.29	4.72e-3	6.90	0.00
Patent-Filing	0.49	7.66e-3	5.85	7.69e-3	5.36	0.00
Regulatory-Investigation-Completed	3.28	6.26e-3	-1.16	1.25e-2	-4.44	0.12

Side-by-side comparison of average abnormal returns (AAR) for the pharmaceutical and biotechnology sectors across the top categories from Table 3, that elicited positive market reactions. Differences in AR are calculated by subtracting pharmaceutical values from biotechnology counterparts. The significance of these differences is determined using a Welch's T-test, with p-values (one-tailed) indicating the likelihood of the results occurring by chance. Categories where the p-value exceeds 5% are marked in red to denote lower statistical significance.

Table S3 (b). Comparative Analysis of Average Abnormal Return (AAR) for Top Negative Abnormal Return (AR) Categories in Pharmaceutical and Biotechnology Sectors Based on Table 3 Data

	Pharmaceutical		Biotechnology		Difference	
Category	AAR (%)	Var	AAR (%)	Var	AR (%)	P-Value
Analyst-Ratings-Negative	-4.66	7.30e-4	-7.31	9.40e-4	-2.65	0.00
Bought-Deal	-5.79	1.37e-2	-9.54	1.07e-2	-3.75	0.13
Clinical-Trials-Negative	-4.29	4.37e-3	-26.03	6.29e-3	-21.74	0.00
Clinical-Trials-Suspended	-1.55	2.81e-3	-14.03	4.64e-3	-12.48	0.00
Earnings-Guidance-Suspended	-6.57	8.97e-3	-13.68	8.16e-3	-7.11	0.01
Expenses-Guidance-Down	-3.25	7.13e-3	-1.41	7.04e-3	1.84	0.16
Operating-Earnings-Negative	-2.73	5.21e-3	-0.65	3.82e-3	2.08	0.02
Patient-Enrollment-Suspended	-8.81	6.58e-3	-10.00	8.53e-3	-1.19	0.34
Product-Discontinued	-1.63	1.69e-3	-9.03	4.37e-3	-7.40	0.00
Product-Outage	-3.33	3.35e-3	-18.88	1.33e-2	-15.55	0.00
Product-Application-Withdrawn	-1.53	4.25e-3	-10.42	7.81e-3	-8.89	0.00
Product-Approval-Denied	-5.81	3.33e-3	-16.26	8.69e-3	-10.45	0.00
Reorganization	-3.01	4.99e-3	-3.45	4.97e-3	-0.44	0.35
Revenue-Guidance-Down	-3.83	2.11e-3	-5.65	2.79e-3	-1.82	0.00
Reverse-Stock-Splits	-10.63	1.05e-2	-8.19	6.10e-3	2.44	0.09

Side-by-side comparison of average abnormal returns (AAR) for the pharmaceutical and biotechnology sectors across the top categories from Table 3, that elicited negative market reactions. Differences in AR are calculated by subtracting pharmaceutical values from biotechnology counterparts. The significance of these differences is determined using a Welch's T-test, with p-values (one-tailed) indicating the likelihood of the results occurring by chance. Categories where the p-value exceeds 5% are marked in red to denote lower statistical significance.
