# Supplementary material for: How does news affect biopharma stock prices?: An event study
Source: PLoS One. 2024 Jan 26;19(1):e0296927. doi: 10.1371/journal.pone.0296927 (PMC10817120; doi:10.1371/journal.pone.0296927)
Supplement: S1 Fig — By broad category, they are (A),(B): acquisition-acquiree, (C),(D): acquisition-acquirer, (E),(F): merger, (G),(H): partnership, and (I),(J): joint-venture. (PDF) [file pone.0296927.s009.pdf]

Supporting Information

Fig S1. Event Study Plots for the Success and Failure of Collaboration-related News

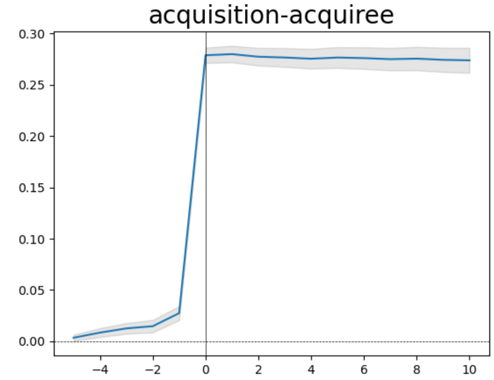

(A)

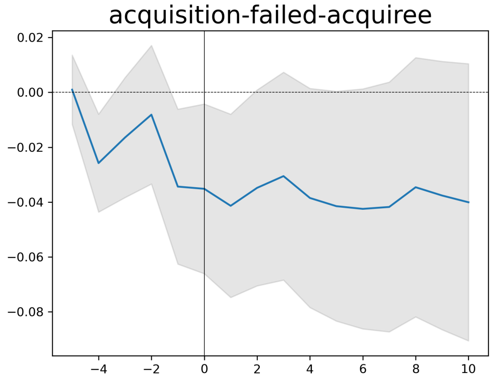

(B)

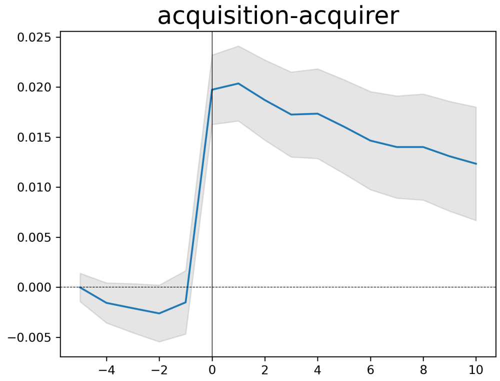

(C)

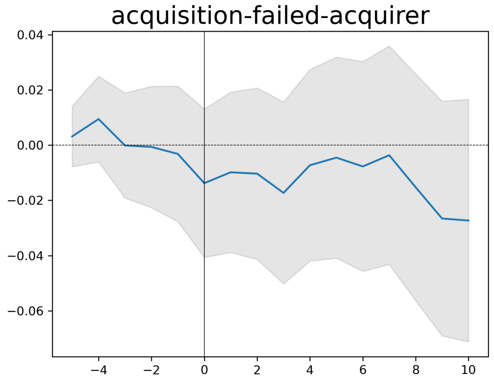

(D)

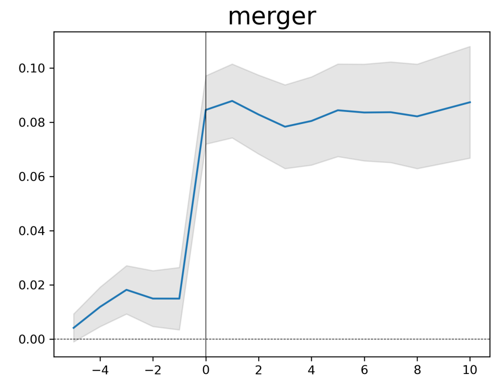

(E)

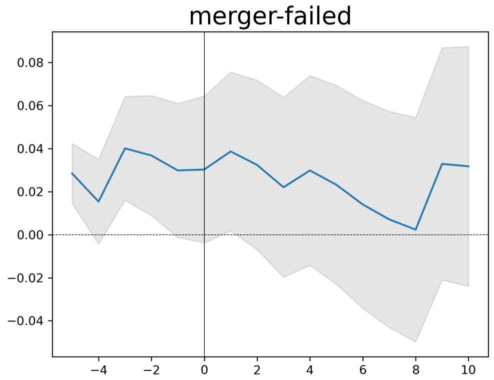

(F)

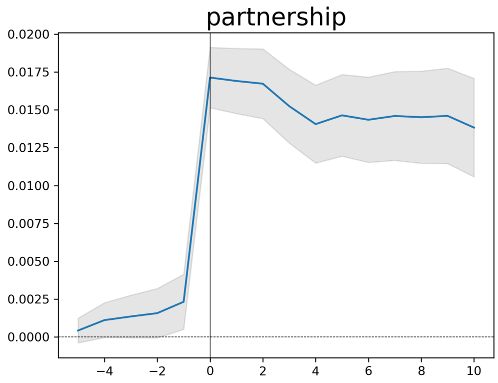
partnership
0.0200
0.0175
0.0150
0.0125
0.0100
0.0075
0.0050
0.0025
0.0000
−4
−2
0
2
4
6
8
10

(G)

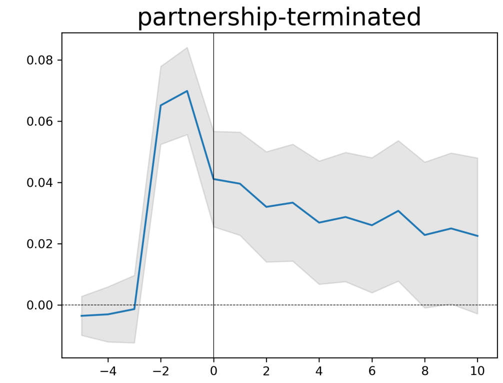
partnership-terminated
0.08
0.06
0.04
0.02
0.00
−4
−2
0
2
4
6
8
10

(H)

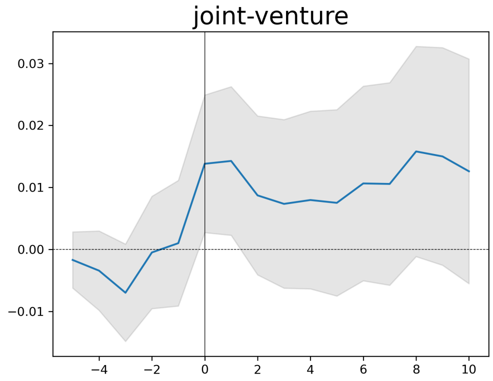
joint-venture
0.03
0.02
0.01
0.00
−0.01
−4
−2
0
2
4
6
8
10

(I)

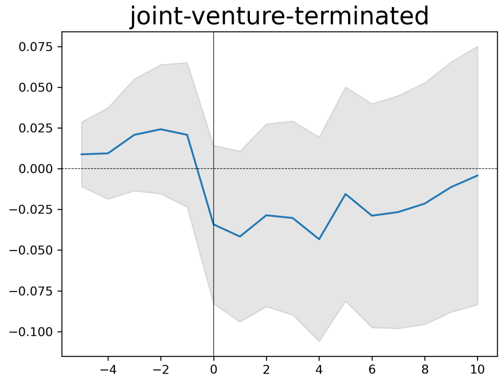
joint-venture-terminated
0.075
0.050
0.025
0.000
−0.025
−0.050
−0.075
−0.100
−4
−2
0
2
4
6
8
10

(J)
