# Supplementary figures and images for: How does news affect biopharma stock prices?: An event study
Source: PLoS One. 2024 Jan 26;19(1):e0296927. doi: 10.1371/journal.pone.0296927 (PMC10817120; doi:10.1371/journal.pone.0296927)

# Supporting Information

**Fig S2. Event Study Plots for Product-Services Related News**

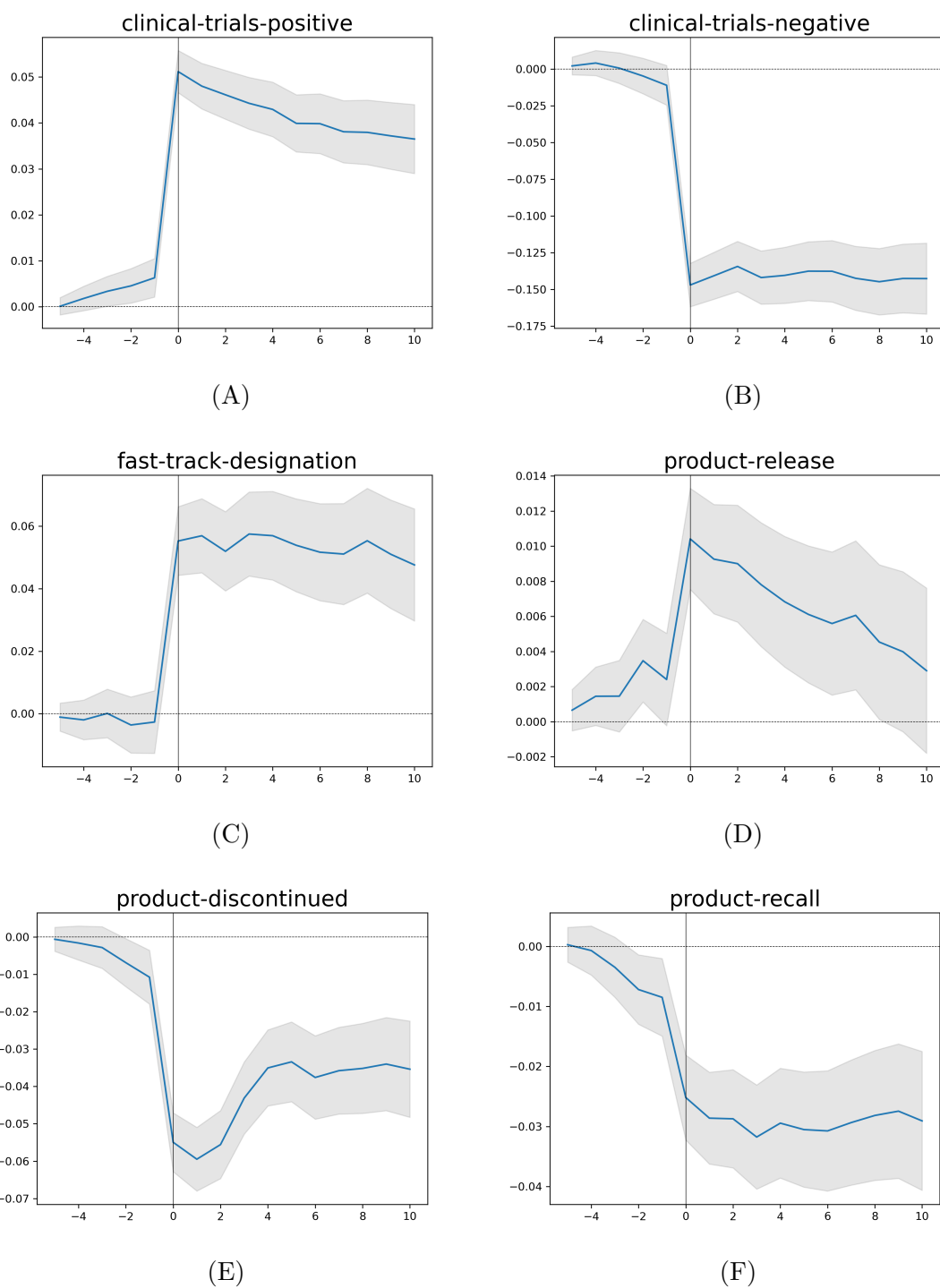

Supplement: S2 Fig — By broad category, they are (A),(B): clinical-trials, (C): fast-track-designation, and (D),(E),(F): product. (PDF) [file pone.0296927.s010.pdf]
